# 242 Accu-Check Glucometer Challenge: Focus on the Prevention of Transmission of Blood-borne Pathogens

**DOI:** 10.1017/ash.2026.10615

**Published:** 2026-06-23

**Authors:** Megan Dethloff, Patrick Reich, Kevin Hsueh, Lydia Grimes-Jenkins, Josephine Fox

**Affiliations:** 1 BJC HealthCare; 2 Washington University School of Medicine; 3 Barnes-Jewish Hospital

## Abstract

**Background:** Candida auris (C. auris) is an emerging pathogen that is often resistant to antifungal treatments and is exceptionally transmissible in the healthcare environment. C. auris colonization confers risk for invasive infections, such as bloodstream infections, surgical site infections and hardware-associated infections. Further, colonization or infection frequently delays or disrupts access to various healthcare venues. Infection prevention (IP) measures including strict contact isolation, specific cleaning and disinfection protocols, and surveillance testing are commonly used strategies to decrease the risk of C. auris transmission. Early identification of patients with C. auris is critical to prevent transmission in the healthcare setting. **Methods:** PCR-based C. auris surveillance testing via surface E-swab of the axillae and inguinal folds was implemented in April 2024 at our large midwestern academic hospital. Testing strategies include Ring Surveillance (targeting patients admitted to adjacent rooms of index patients with C. auris), Travel Screening (i.e., recent hospitalization outside the U.S.), and long-term accute care (LTAC) Screening (targeting recent admission to a long-term acute care or inpatient rehabilitation/nursing facility). A retrospective review of adult patients who underwent C. auris surveillance testing between May 1, 2025 – October 31, 2025, was performed. Patient demographics (sex, race, ethnicity, mean age at admission) were analyzed along with admission source, comorbidities, and admission diagnosis. Patients with positive results were compared based on testing indication; specifically, those tested as part of Ring Surveillance and those tested on admission due to Travel or LTAC Screening. **Results:** In the 6-month study period, 4624 patients were tested for C. auris and only 170 (0.6%) had positive results; 118 through ring surveillance and 52 found with an admission testing indication. Positive patients had a mean age was 59 years old and 67% identified as male. 73.5% of positive patients were hospitalized within the last 6 months. **Conclusions:** Prevention of C. auris transmission in the healthcare setting remains an IP challenge. The prevalence of C. auris was low in our cohort of patients who were primarily tested as part of a Ring Surveillance program. Development of a predictive model to optimize early identification of patients colonized with C. auris is important to guide surveillance testing and decrease transmission risk to other patients in the healthcare setting.

